# 1-Benzoyl-*c*-3,*t*-3-dimethyl-*r*-2,*c*-6-diphenyl­piperidin-4-one

**DOI:** 10.1107/S1600536809028037

**Published:** 2009-07-25

**Authors:** S. Aravindhan, S. Ponnuswamy, J. Umamaheswari, P. Ramesh, M. N. Ponnuswamy

**Affiliations:** a Department of Physics, Presidency College (Autonomous), Chennai 600 005, India; b Department of Chemistry, Government Arts College (Autonomous), Coimbatore 641 018, India; c Centre of Advanced Study in Crystallography and Biophysics, University of Madras, Guindy Campus, Chennai 600 025, India

## Abstract

In the title compound, C_26_H_25_NO_2_, the piperidine ring adopts a distorted boat conformation. The three phenyl rings form dihedral angles of 67.58 (8), 59.82 (8) and 86.41 (8)° with the best plane through the piperidine ring. The crystal packing is governed by inter­molecular C—H⋯O inter­actions.

## Related literature

For the biological activity of piperidine derivatives, see: Dimmock *et al.* (2001 ▶); Perumal *et al.* (2001 ▶). For hydrogen-bond motifs, see: Bernstein *et al.* (1995 ▶). For puckering and asymmetry parameters, see: Cremer & Pople (1975 ▶); Nardelli (1983 ▶).
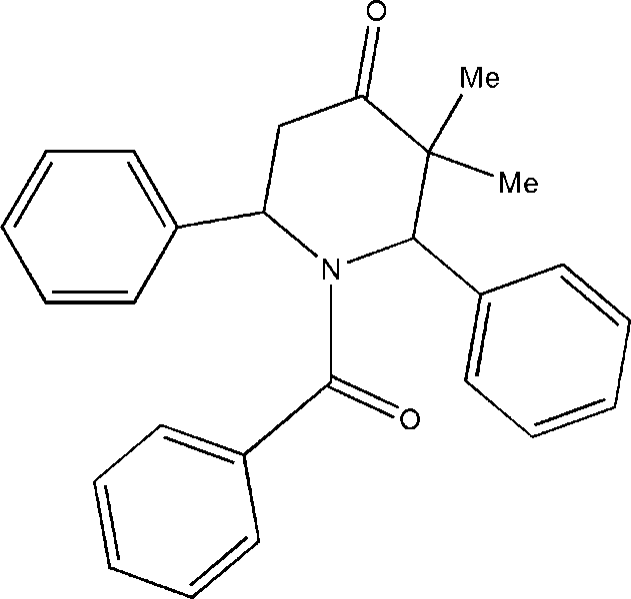


## Experimental

### 

#### Crystal data

C_26_H_25_NO_2_
*M* *_r_* = 383.47
Monoclinic, 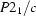

*a* = 10.8540 (9) Å
*b* = 17.8050 (17) Å
*c* = 10.8853 (10) Å
β = 94.987 (3)°
*V* = 2095.7 (3) Å^3^
*Z* = 4
Mo *K*α radiation
μ = 0.08 mm^−1^
*T* = 293 K
0.30 × 0.25 × 0.20 mm

#### Data collection

Bruker Kappa APEXII area-detector diffractometer
Absorption correction: multi-scan (*SADABS*; Sheldrick, 2001 ▶) *T*_min_ = 0.977, *T*_max_ = 0.985
27356 measured reflections
6189 independent reflections
3897 reflections with *I* > 2σ(*I*)
*R*_int_ = 0.038

#### Refinement

*R*[*F* ^2^ > 2σ(*F* ^2^)] = 0.050
*wR*(*F* ^2^) = 0.152
*S* = 0.98
6189 reflections
265 parameters
H-atom parameters constrained
Δρ_max_ = 0.24 e Å^−3^
Δρ_min_ = −0.17 e Å^−3^

### 

Data collection: *APEX2* (Bruker, 2004 ▶); cell refinement: *SAINT* (Bruker, 2004 ▶); data reduction: *SAINT*; program(s) used to solve structure: *SHELXS97* (Sheldrick, 2008 ▶); program(s) used to refine structure: *SHELXL97* (Sheldrick, 2008 ▶); molecular graphics: *ORTEP-3* (Farrugia, 1997 ▶); software used to prepare material for publication: *SHELXL97* and *PLATON* (Spek, 2009 ▶).

## Supplementary Material

Crystal structure: contains datablocks global, I. DOI: 10.1107/S1600536809028037/bt2990sup1.cif

Structure factors: contains datablocks I. DOI: 10.1107/S1600536809028037/bt2990Isup2.hkl

Additional supplementary materials:  crystallographic information; 3D view; checkCIF report

## Figures and Tables

**Table 1:** Hydrogen-bond geometry (Å, °)

*D*—H⋯*A*	*D*—H	H⋯*A*	*D*⋯*A*	*D*—H⋯*A*
C6—H6⋯O1	0.98	2.29	2.7346 (17)	106
C2—H2⋯O1^i^	0.98	2.56	3.3784 (17)	141
C20—H20*A*⋯O1^i^	0.96	2.47	3.1885 (19)	132
C20—H20*B*⋯O2^ii^	0.96	2.52	3.470 (2)	170

Symmetry codes: (i) 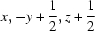; (ii) 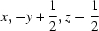.

**Fig. 1.**
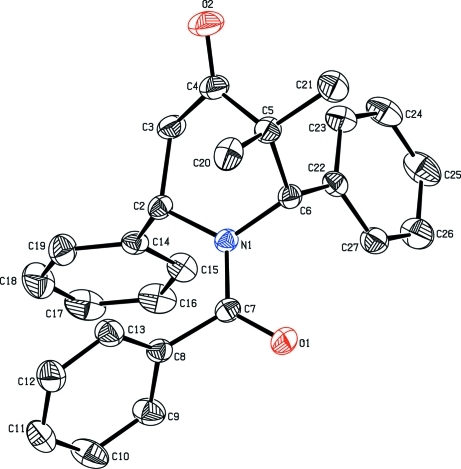
Perspective view of the molecule showing displacement ellipsoids at 50% probability level. The H atoms are omitted for clarity.

**Fig. 2.**
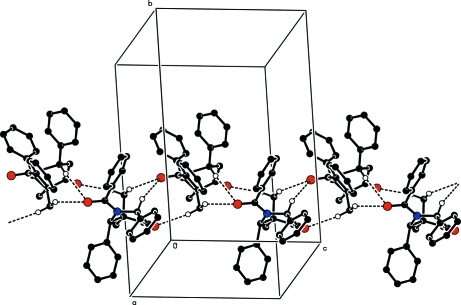
The crystal packing viewed down a axis. H atoms not involved in hydrogen bonding have been omitted for clarity.

## supplementary crystallographic information

### Comment

Piperidones are the important group of heterocyclic compounds in the field of medicinal chemistry due to their biological activities, including cytotoxic and anticancer properties (Dimmock *et al.*, 2001). They were also reported to possess analgesic, anti-inflammatory, central nervous system (CNS), local anaesthetic, anticancer and antimicrobial activities (Perumal *et al.*, 2001). In view of these importance and to ascertain the molecular conformation, crystallographic study of the title compound has been carried out.

The *ORTEP* diagram of the title compound is shown in Fig.1. The piperidine ring adopts distorted boat conformation. The puckering parameters (Cremer & Pople, 1975) and the asymmetry parameters (Nardelli, 1983) for this ring are q_2_ = 0.636 (2) Å, q_3_ = 0.104 (2) Å, π = 282.8 (1)° and Δs(C3) =Δs(C6)= 18.6 (1)°. The sum of the angles at N1 (359.7°) is in accrdance with *sp*^2^ hybridization. The three phenyl rings are twisted away from the best plane of the pyridine ring by 67.58 (8)°, 59.82 (8)° and 86.41 (8)° respectively.

The crystal packing is controlled by C—H···O types of intra and intermolecular interactions in addition to van der Waals forces. Atom C2 at (*x*, *y*, *z*) donates a proton to O1 *x*, -*y* + 1/2, *z* + 1/2, which forms a C(5) (Bernstein, *et al.*, 1995) zigzag chain running along *c* axis. The combination of C20—H20A···O1 and C20—H20B···O2 intermolecular interactions forms a dimer chain running along *c* axis shown in Fig. 2.

### Experimental

A mixture of c-3,t-3-dimethyl-r-2,c-6-diphenylpiperidin-4-one (1.4 g, 5 mmol), benzoyl chloride (1.2 ml, 10 mmol) and triethylamine (2 ml, 14.4 mmol) in anhydrous benzene (20 ml) was stirred at room temperature for 7 h. The precipitated ammonium salt was washed with water (4x10ml). The resulting pasty mass was purified and crystallized from benzene and pet-ether (60–80°C) in the ratio of 95: 5.

### Refinement

All H atoms were positioned geometrically (C—H=0.93–0.98 Å) and allowed to ride on their parent atoms, with 1.5*U*_eq_(C) for methyl H and 1.2 *U*_eq_(C) for other H atoms.

### Crystal data

**Table:**

C_26_H_25_NO_2_	*F*(000) = 816
*M**_r_* = 383.47	*D*_x_ = 1.215 Mg m^−^^3^
Monoclinic, *P*2_1_/*c*	Mo *K*α radiation, λ = 0.71073 Å
Hall symbol: -P 2ybc	Cell parameters from 5746 reflections
*a* = 10.8540 (9) Å	θ = 1.9–30.4°
*b* = 17.8050 (17) Å	µ = 0.08 mm^−^^1^
*c* = 10.8853 (10) Å	*T* = 293 K
β = 94.987 (3)°	Block, colorless
*V* = 2095.7 (3) Å^3^	0.30 × 0.25 × 0.20 mm
*Z* = 4	

### Data collection

**Table:**

Bruker Kappa APEXII area-detector diffractometer	6189 independent reflections
Radiation source: fine-focus sealed tube	3897 reflections with *I* > 2σ(*I*)
graphite	*R*_int_ = 0.038
ω and φ scans	θ_max_ = 30.4°, θ_min_ = 1.9°
Absorption correction: multi-scan (*SADABS*; Sheldrick, 2001)	*h* = −15→13
*T*_min_ = 0.977, *T*_max_ = 0.985	*k* = −25→25
27356 measured reflections	*l* = −15→15

### Refinement

**Table:**

Refinement on *F*^2^	Secondary atom site location: difference Fourier map
Least-squares matrix: full	Hydrogen site location: inferred from neighbouring sites
*R*[*F*^2^ > 2σ(*F*^2^)] = 0.050	H-atom parameters constrained
*wR*(*F*^2^) = 0.152	*w* = 1/[σ^2^(*F*_o_^2^) + (0.0728*P*)^2^ + 0.3353*P*] where *P* = (*F*_o_^2^ + 2*F*_c_^2^)/3
*S* = 0.98	(Δ/σ)_max_ = 0.011
6189 reflections	Δρ_max_ = 0.24 e Å^−^^3^
265 parameters	Δρ_min_ = −0.17 e Å^−^^3^
0 restraints	Extinction correction: *SHELXL97* (Sheldrick, 2008), Fc^*^=kFc[1+0.001xFc^2^λ^3^/sin(2θ)]^-1/4^
Primary atom site location: structure-invariant direct methods	Extinction coefficient: 0.0078 (16)

### Special details

**Table:**

Geometry. All e.s.d.'s (except the e.s.d. in the dihedral angle between two l.s. planes) are estimated using the full covariance matrix. The cell e.s.d.'s are taken into account individually in the estimation of e.s.d.'s in distances, angles and torsion angles; correlations between e.s.d.'s in cell parameters are only used when they are defined by crystal symmetry. An approximate (isotropic) treatment of cell e.s.d.'s is used for estimating e.s.d.'s involving l.s. planes.
Refinement. Refinement of *F*^2^ against ALL reflections. The weighted *R*-factor *wR* and goodness of fit *S* are based on *F*^2^, conventional *R*-factors *R* are based on *F*, with *F* set to zero for negative *F*^2^. The threshold expression of *F*^2^ > σ(*F*^2^) is used only for calculating *R*-factors(gt) *etc*. and is not relevant to the choice of reflections for refinement. *R*-factors based on *F*^2^ are statistically about twice as large as those based on *F*, and *R*- factors based on ALL data will be even larger.

### Fractional atomic coordinates and isotropic or equivalent isotropic displacement parameters (Å^2^)

**Table:**

	*x*	*y*	*z*	*U*_iso_*/*U*_eq_	
O1	0.14493 (11)	0.19520 (7)	0.50443 (9)	0.0560 (3)	
O2	0.41301 (12)	0.16165 (9)	1.02065 (10)	0.0722 (4)	
N1	0.17515 (10)	0.16380 (6)	0.70591 (9)	0.0338 (2)	
C2	0.12064 (12)	0.15019 (8)	0.82403 (11)	0.0361 (3)	
H2	0.0881	0.1980	0.8520	0.043*	
C3	0.21997 (14)	0.12271 (9)	0.92293 (12)	0.0466 (4)	
H3A	0.1886	0.1294	1.0030	0.056*	
H3B	0.2313	0.0692	0.9113	0.056*	
C4	0.34508 (15)	0.15986 (10)	0.92630 (13)	0.0493 (4)	
C5	0.38114 (14)	0.19299 (9)	0.80660 (13)	0.0446 (3)	
C6	0.30890 (12)	0.15316 (7)	0.69712 (11)	0.0362 (3)	
H6	0.3282	0.1809	0.6235	0.043*	
C7	0.10627 (13)	0.19291 (8)	0.60735 (11)	0.0373 (3)	
C8	−0.01836 (13)	0.22524 (8)	0.62390 (11)	0.0376 (3)	
C9	−0.12156 (16)	0.19732 (9)	0.55574 (16)	0.0543 (4)	
H9	−0.1149	0.1550	0.5067	0.065*	
C10	−0.23461 (17)	0.23218 (11)	0.5604 (2)	0.0681 (5)	
H10	−0.3040	0.2125	0.5155	0.082*	
C11	−0.24602 (16)	0.29517 (10)	0.62980 (18)	0.0600 (4)	
H11	−0.3223	0.3187	0.6312	0.072*	
C12	−0.14437 (17)	0.32334 (10)	0.69727 (17)	0.0588 (4)	
H12	−0.1516	0.3664	0.7445	0.071*	
C13	−0.03124 (15)	0.28842 (9)	0.69574 (15)	0.0507 (4)	
H13	0.0370	0.3075	0.7433	0.061*	
C14	0.01468 (13)	0.09444 (8)	0.80976 (12)	0.0385 (3)	
C15	0.01631 (15)	0.03311 (8)	0.73135 (15)	0.0485 (4)	
H15	0.0832	0.0259	0.6848	0.058*	
C16	−0.08083 (17)	−0.01740 (10)	0.72184 (18)	0.0608 (5)	
H16	−0.0791	−0.0582	0.6688	0.073*	
C17	−0.18017 (18)	−0.00735 (12)	0.7908 (2)	0.0699 (5)	
H17	−0.2455	−0.0413	0.7844	0.084*	
C18	−0.18221 (18)	0.05259 (13)	0.8683 (2)	0.0724 (6)	
H18	−0.2490	0.0592	0.9152	0.087*	
C19	−0.08585 (15)	0.10376 (10)	0.87796 (15)	0.0561 (4)	
H19	−0.0888	0.1447	0.9307	0.067*	
C20	0.34155 (16)	0.27631 (9)	0.80821 (15)	0.0539 (4)	
H20A	0.2550	0.2794	0.8196	0.081*	
H20B	0.3569	0.2995	0.7314	0.081*	
H20C	0.3882	0.3018	0.8747	0.081*	
C21	0.52053 (15)	0.19025 (12)	0.79462 (18)	0.0646 (5)	
H21A	0.5634	0.2149	0.8642	0.097*	
H21B	0.5386	0.2153	0.7202	0.097*	
H21C	0.5470	0.1389	0.7920	0.097*	
C22	0.34321 (13)	0.07146 (8)	0.67344 (13)	0.0397 (3)	
C23	0.40686 (15)	0.02446 (10)	0.75922 (15)	0.0532 (4)	
H23	0.4300	0.0424	0.8381	0.064*	
C24	0.43630 (17)	−0.04821 (10)	0.72953 (19)	0.0638 (5)	
H24	0.4789	−0.0787	0.7882	0.077*	
C25	0.40313 (18)	−0.07557 (10)	0.6143 (2)	0.0700 (5)	
H25	0.4234	−0.1246	0.5943	0.084*	
C26	0.33977 (17)	−0.03070 (10)	0.52792 (18)	0.0627 (5)	
H26	0.3162	−0.0495	0.4497	0.075*	
C27	0.31101 (14)	0.04226 (9)	0.55720 (14)	0.0475 (4)	
H27	0.2691	0.0724	0.4976	0.057*	

### Atomic displacement parameters (Å^2^)

**Table:**

	*U*^11^	*U*^22^	*U*^33^	*U*^12^	*U*^13^	*U*^23^
O1	0.0589 (7)	0.0747 (8)	0.0352 (5)	0.0155 (6)	0.0096 (5)	0.0121 (5)
O2	0.0575 (8)	0.1115 (11)	0.0444 (6)	−0.0127 (7)	−0.0138 (5)	−0.0013 (6)
N1	0.0348 (6)	0.0364 (6)	0.0304 (5)	−0.0024 (4)	0.0035 (4)	0.0021 (4)
C2	0.0398 (7)	0.0379 (7)	0.0308 (6)	−0.0046 (6)	0.0045 (5)	0.0026 (5)
C3	0.0469 (9)	0.0608 (9)	0.0314 (6)	−0.0062 (7)	−0.0002 (6)	0.0079 (6)
C4	0.0465 (9)	0.0623 (10)	0.0377 (7)	−0.0050 (7)	−0.0046 (6)	−0.0025 (6)
C5	0.0382 (8)	0.0535 (8)	0.0418 (7)	−0.0107 (6)	0.0023 (6)	−0.0053 (6)
C6	0.0346 (7)	0.0400 (7)	0.0342 (6)	−0.0031 (5)	0.0044 (5)	0.0009 (5)
C7	0.0424 (8)	0.0365 (7)	0.0329 (6)	−0.0008 (6)	0.0025 (5)	0.0033 (5)
C8	0.0400 (8)	0.0384 (7)	0.0341 (6)	0.0001 (6)	0.0012 (5)	0.0054 (5)
C9	0.0520 (10)	0.0482 (9)	0.0602 (9)	0.0012 (7)	−0.0090 (7)	−0.0065 (7)
C10	0.0455 (10)	0.0635 (11)	0.0914 (13)	−0.0029 (8)	−0.0171 (9)	−0.0044 (10)
C11	0.0421 (10)	0.0613 (11)	0.0772 (11)	0.0092 (8)	0.0096 (8)	0.0086 (9)
C12	0.0585 (11)	0.0559 (10)	0.0623 (10)	0.0107 (8)	0.0065 (8)	−0.0072 (8)
C13	0.0465 (9)	0.0514 (9)	0.0530 (8)	0.0019 (7)	−0.0029 (7)	−0.0096 (7)
C14	0.0380 (8)	0.0398 (7)	0.0372 (6)	−0.0028 (6)	−0.0002 (5)	0.0102 (5)
C15	0.0461 (9)	0.0402 (8)	0.0588 (9)	−0.0051 (6)	0.0020 (7)	0.0019 (6)
C16	0.0605 (11)	0.0441 (9)	0.0748 (11)	−0.0121 (8)	−0.0106 (9)	0.0065 (8)
C17	0.0543 (11)	0.0667 (12)	0.0860 (13)	−0.0249 (9)	−0.0094 (9)	0.0264 (10)
C18	0.0478 (11)	0.0892 (15)	0.0825 (13)	−0.0161 (10)	0.0185 (9)	0.0165 (11)
C19	0.0482 (10)	0.0659 (11)	0.0556 (9)	−0.0082 (8)	0.0125 (7)	0.0015 (8)
C20	0.0578 (10)	0.0517 (9)	0.0534 (9)	−0.0181 (8)	0.0115 (7)	−0.0115 (7)
C21	0.0408 (10)	0.0849 (13)	0.0679 (11)	−0.0175 (9)	0.0028 (8)	−0.0122 (9)
C22	0.0322 (7)	0.0419 (7)	0.0450 (7)	−0.0006 (6)	0.0034 (5)	0.0021 (6)
C23	0.0476 (9)	0.0557 (9)	0.0546 (9)	0.0042 (7)	−0.0047 (7)	0.0055 (7)
C24	0.0506 (10)	0.0528 (10)	0.0855 (13)	0.0092 (8)	−0.0085 (9)	0.0137 (9)
C25	0.0571 (12)	0.0432 (9)	0.1068 (15)	0.0095 (8)	−0.0094 (10)	−0.0086 (10)
C26	0.0609 (11)	0.0520 (10)	0.0728 (11)	0.0076 (8)	−0.0074 (9)	−0.0174 (8)
C27	0.0454 (9)	0.0468 (8)	0.0493 (8)	0.0055 (7)	−0.0021 (6)	−0.0041 (6)

### Geometric parameters (Å, °)

**Table:**

O1—C7	1.2307 (16)	C14—C19	1.382 (2)
O2—C4	1.2115 (17)	C14—C15	1.387 (2)
N1—C7	1.3561 (16)	C15—C16	1.383 (2)
N1—C6	1.4754 (17)	C15—H15	0.9300
N1—C2	1.4813 (15)	C16—C17	1.378 (3)
C2—C14	1.5170 (19)	C16—H16	0.9300
C2—C3	1.5359 (19)	C17—C18	1.362 (3)
C2—H2	0.9800	C17—H17	0.9300
C3—C4	1.508 (2)	C18—C19	1.384 (3)
C3—H3A	0.9700	C18—H18	0.9300
C3—H3B	0.9700	C19—H19	0.9300
C4—C5	1.513 (2)	C20—H20A	0.9600
C5—C21	1.531 (2)	C20—H20B	0.9600
C5—C6	1.5412 (19)	C20—H20C	0.9600
C5—C20	1.545 (2)	C21—H21A	0.9600
C6—C22	1.529 (2)	C21—H21B	0.9600
C6—H6	0.9800	C21—H21C	0.9600
C7—C8	1.495 (2)	C22—C27	1.384 (2)
C8—C9	1.381 (2)	C22—C23	1.392 (2)
C8—C13	1.384 (2)	C23—C24	1.378 (2)
C9—C10	1.380 (3)	C23—H23	0.9300
C9—H9	0.9300	C24—C25	1.364 (3)
C10—C11	1.364 (3)	C24—H24	0.9300
C10—H10	0.9300	C25—C26	1.372 (3)
C11—C12	1.367 (3)	C25—H25	0.9300
C11—H11	0.9300	C26—C27	1.380 (2)
C12—C13	1.378 (2)	C26—H26	0.9300
C12—H12	0.9300	C27—H27	0.9300
C13—H13	0.9300		
			
C7—N1—C6	118.40 (10)	C8—C13—H13	119.7
C7—N1—C2	120.99 (11)	C19—C14—C15	118.55 (14)
C6—N1—C2	120.31 (10)	C19—C14—C2	119.56 (13)
N1—C2—C14	112.00 (10)	C15—C14—C2	121.88 (13)
N1—C2—C3	110.59 (11)	C16—C15—C14	120.51 (16)
C14—C2—C3	110.06 (11)	C16—C15—H15	119.7
N1—C2—H2	108.0	C14—C15—H15	119.7
C14—C2—H2	108.0	C17—C16—C15	120.16 (18)
C3—C2—H2	108.0	C17—C16—H16	119.9
C4—C3—C2	116.94 (12)	C15—C16—H16	119.9
C4—C3—H3A	108.1	C18—C17—C16	119.66 (17)
C2—C3—H3A	108.1	C18—C17—H17	120.2
C4—C3—H3B	108.1	C16—C17—H17	120.2
C2—C3—H3B	108.1	C17—C18—C19	120.68 (18)
H3A—C3—H3B	107.3	C17—C18—H18	119.7
O2—C4—C3	120.83 (14)	C19—C18—H18	119.7
O2—C4—C5	122.41 (15)	C14—C19—C18	120.43 (17)
C3—C4—C5	116.74 (12)	C14—C19—H19	119.8
C4—C5—C21	113.12 (14)	C18—C19—H19	119.8
C4—C5—C6	109.53 (12)	C5—C20—H20A	109.5
C21—C5—C6	111.08 (12)	C5—C20—H20B	109.5
C4—C5—C20	105.76 (12)	H20A—C20—H20B	109.5
C21—C5—C20	108.03 (13)	C5—C20—H20C	109.5
C6—C5—C20	109.10 (12)	H20A—C20—H20C	109.5
N1—C6—C22	112.86 (11)	H20B—C20—H20C	109.5
N1—C6—C5	109.16 (10)	C5—C21—H21A	109.5
C22—C6—C5	116.95 (12)	C5—C21—H21B	109.5
N1—C6—H6	105.6	H21A—C21—H21B	109.5
C22—C6—H6	105.6	C5—C21—H21C	109.5
C5—C6—H6	105.6	H21A—C21—H21C	109.5
O1—C7—N1	121.65 (13)	H21B—C21—H21C	109.5
O1—C7—C8	118.71 (12)	C27—C22—C23	117.29 (14)
N1—C7—C8	119.61 (11)	C27—C22—C6	117.74 (12)
C9—C8—C13	118.61 (14)	C23—C22—C6	124.95 (13)
C9—C8—C7	119.78 (13)	C24—C23—C22	121.26 (16)
C13—C8—C7	121.19 (13)	C24—C23—H23	119.4
C10—C9—C8	120.05 (16)	C22—C23—H23	119.4
C10—C9—H9	120.0	C25—C24—C23	120.19 (16)
C8—C9—H9	120.0	C25—C24—H24	119.9
C11—C10—C9	120.93 (17)	C23—C24—H24	119.9
C11—C10—H10	119.5	C24—C25—C26	119.90 (17)
C9—C10—H10	119.5	C24—C25—H25	120.0
C10—C11—C12	119.45 (16)	C26—C25—H25	120.0
C10—C11—H11	120.3	C25—C26—C27	119.99 (17)
C12—C11—H11	120.3	C25—C26—H26	120.0
C11—C12—C13	120.44 (16)	C27—C26—H26	120.0
C11—C12—H12	119.8	C26—C27—C22	121.36 (15)
C13—C12—H12	119.8	C26—C27—H27	119.3
C12—C13—C8	120.50 (15)	C22—C27—H27	119.3
C12—C13—H13	119.7		
			
C7—N1—C2—C14	61.05 (16)	C13—C8—C9—C10	−0.2 (2)
C6—N1—C2—C14	−125.24 (12)	C7—C8—C9—C10	−172.85 (15)
C7—N1—C2—C3	−175.79 (12)	C8—C9—C10—C11	1.3 (3)
C6—N1—C2—C3	−2.08 (16)	C9—C10—C11—C12	−1.1 (3)
N1—C2—C3—C4	40.28 (18)	C10—C11—C12—C13	−0.2 (3)
C14—C2—C3—C4	164.56 (13)	C11—C12—C13—C8	1.3 (3)
C2—C3—C4—O2	154.70 (16)	C9—C8—C13—C12	−1.1 (2)
C2—C3—C4—C5	−26.3 (2)	C7—C8—C13—C12	171.44 (14)
O2—C4—C5—C21	30.2 (2)	N1—C2—C14—C19	−144.35 (13)
C3—C4—C5—C21	−148.72 (15)	C3—C2—C14—C19	92.20 (16)
O2—C4—C5—C6	154.74 (16)	N1—C2—C14—C15	36.78 (18)
C3—C4—C5—C6	−24.22 (19)	C3—C2—C14—C15	−86.67 (15)
O2—C4—C5—C20	−87.8 (2)	C19—C14—C15—C16	0.1 (2)
C3—C4—C5—C20	93.23 (16)	C2—C14—C15—C16	178.96 (13)
C7—N1—C6—C22	−102.18 (13)	C14—C15—C16—C17	−0.3 (2)
C2—N1—C6—C22	83.95 (14)	C15—C16—C17—C18	0.0 (3)
C7—N1—C6—C5	125.98 (13)	C16—C17—C18—C19	0.4 (3)
C2—N1—C6—C5	−47.89 (15)	C15—C14—C19—C18	0.3 (2)
C4—C5—C6—N1	59.99 (15)	C2—C14—C19—C18	−178.58 (16)
C21—C5—C6—N1	−174.33 (13)	C17—C18—C19—C14	−0.6 (3)
C20—C5—C6—N1	−55.34 (14)	N1—C6—C22—C27	72.51 (15)
C4—C5—C6—C22	−69.65 (16)	C5—C6—C22—C27	−159.62 (13)
C21—C5—C6—C22	56.03 (17)	N1—C6—C22—C23	−108.93 (15)
C20—C5—C6—C22	175.02 (11)	C5—C6—C22—C23	18.9 (2)
C6—N1—C7—O1	16.05 (19)	C27—C22—C23—C24	−0.1 (2)
C2—N1—C7—O1	−170.12 (13)	C6—C22—C23—C24	−178.70 (15)
C6—N1—C7—C8	−162.10 (11)	C22—C23—C24—C25	0.0 (3)
C2—N1—C7—C8	11.73 (18)	C23—C24—C25—C26	−0.3 (3)
O1—C7—C8—C9	59.09 (19)	C24—C25—C26—C27	0.8 (3)
N1—C7—C8—C9	−122.69 (15)	C25—C26—C27—C22	−1.0 (3)
O1—C7—C8—C13	−113.39 (16)	C23—C22—C27—C26	0.6 (2)
N1—C7—C8—C13	64.83 (18)	C6—C22—C27—C26	179.29 (15)

### Hydrogen-bond geometry (Å, °)

**Table:**

*D*—H···*A*	*D*—H	H···*A*	*D*···*A*	*D*—H···*A*
C6—H6···O1	0.98	2.29	2.7346 (17)	106
C2—H2···O1^i^	0.98	2.56	3.3784 (17)	141
C20—H20A···O1^i^	0.96	2.47	3.1885 (19)	132
C20—H20B···O2^ii^	0.96	2.52	3.470 (2)	170

Symmetry codes:  (i) *x*, −*y*+1/2, *z*+1/2; (ii) *x*, −*y*+1/2, *z*−1/2.
